# Neurological characterization of mice deficient in GSK3α highlight pleiotropic physiological functions in cognition and pathological activity as Tau kinase

**DOI:** 10.1186/1756-6606-6-27

**Published:** 2013-05-25

**Authors:** Hervé Maurin, Benoit Lechat, Ilse Dewachter, Laurence Ris, Justin V Louis, Peter Borghgraef, Herman Devijver, Tomasz Jaworski, Fred Van Leuven

**Affiliations:** 1 Experimental Genetics Group - LEGTEGG, Department Human Genetics, KULeuven, B-3000, Leuven, Belgium; 2 Department Neurosciences, University Mons-Hainaut, B-7000, Mons, Belgium; 3 Present address: Nencki Institute Experimental Biology, 3 Pasteur Street, 02-093, Warsaw, Poland

**Keywords:** GSK3α knock-out, Cognition, LTP, Protein Tau, Hippocampus, Motor behavior

## Abstract

**Background:**

GSK3β is involved in a wide range of physiological functions, and is presumed to act in the pathogenesis of neurological diseases, from bipolar disorder to Alzheimer’s disease (AD). In contrast, the GSK3α isozyme remained largely ignored with respect to both aspects.

**Results:**

We generated and characterized two mouse strains with neuron-specific or with total GSK3α deficiency. Behavioral and electrophysiological analysis demonstrated the physiological importance of neuronal GSK3α, with GSK3β not compensating for impaired cognition and reduced LTP. Interestingly, the passive inhibitory avoidance task proved to modulate the phosphorylation status of both GSK3 isozymes in wild-type mice, further implying both to function in cognition. Moreover, GSK3α contributed to the neuronal architecture of the hippocampal CA1 sub-region that is most vulnerable in AD. Consequently, practically all parameters and characteristics indicated that both GSK3 isoforms were regulated independently, but that they acted on the same physiological functions in learning and memory, in mobility and in behavior.

**Conclusions:**

GSK3α proved to be regulated independently from GSK3β, and to exert non-redundant physiological neurological functions in general behavior and in cognition. Moreover, GSK3α contributes to the pathological phosphorylation of protein Tau.

## Background

Glycogen synthase kinase-3 (GSK3) comprises two structurally and functionally related serine-threonine kinases, active in many physiological processes [1-5]. Both are inherently active and controlled by phosphorylation at two levels: (i) inhibitory phosphorylation of serine residues S21/S9 in GSK3α/β and (ii) tyrosine phosphorylation at Y279/Y216 in GSK3α/β, which augments their activity and relieves substrate-priming by other kinases [6,7]. Tyrosine phosphorylation appears an intramolecular autocatalytic event during synthesis and folding, which makes GSK3 dual-specificity kinases. Consequently, each isozyme exists in four different phosphorylated isoforms, a molecular complexity that yet escapes analysis [5]. Combined with expression of both isozymes in most cells, and the wide diversity of substrates and molecular partners, complicates the estimation of activity and definition of functions in vivo.

Neurobiological focus on GSK3β stems from its demonstrated functions in neuronal differentiation and in cognition, and from its role as “tau-kinase I” in Alzheimer’s disease (AD) [8]. GSK3β was proposed as a therapeutic target based on the treatment of bipolar disorder with lithium salts, but this however seriously suffers from limited effectiveness, narrow therapeutic window and side-effects. Moreover, the mode of action of lithium ions is not understood, because they are neither very effective nor specific inhibitors of GSK3α/β [9,10]. Experimental evidence, and localization in dendritic spines [11] supports a post-synaptic role for GSK3β in LTP, and by extension in synaptic plasticity [5,12-17]. Pre-synaptically, GSK3 controls activity-dependent bulk endocytosis [18,19].

The structural similar kinase domains predict that GSK3 isozymes share physiological functions. Nevertheless, GSK3β deficient mice die in utero [20] in contrast with viability of GSK3α deficient mice [21]. This extreme difference in outcome demonstrates their non-redundant physiological functions, which still need to be detailed in vivo.

Neurobiological analysis of GSK3α, particularly in AD remains fragmented and debated [22-25]. The contribution of GSK3β to phosphorylation of protein Tau is evident [26-29] while that of GSK3α is hardly investigated.

In these perspectives, we studied GSK3α in brain in vivo, in two newly generated mouse strains deficient in GSK3α, either completely in all organs or neuron-specific. This effort allowed us to investigate physiological functions and pathological roles, whereby we concentrated on the neurobiological aspects to highlight the physiological functions of GSK3α in learning and memory, in mobility and behavior. In line with their independent regulation and non-redundancy, both GSK3α and GSK3β contributed to the physiological and to the pathological phosphorylation of protein Tau.

## Results

### Generation of two mouse strains with either neuron-specific or with total deficiency of GSK3α

To define the physiological functions of GSK3α in adult brain in vivo, we aimed in first instance to generate mice with a conditional, post-natal and neuron-specific deficiency of GSK3α by the Cre-Lox system. We thereby anticipated to circumvent peripheral problems caused by GSK3α deficiency in peripheral systems. Mice with floxed GSK3α genes [30] were mated with transgenic mice that express Cre-recombinase under control of the mouse Thy1 gene promoter, which we validated previously for post-natal neuronal inactivation of the Presenilin-1 gene [31].

**Figure 1:**
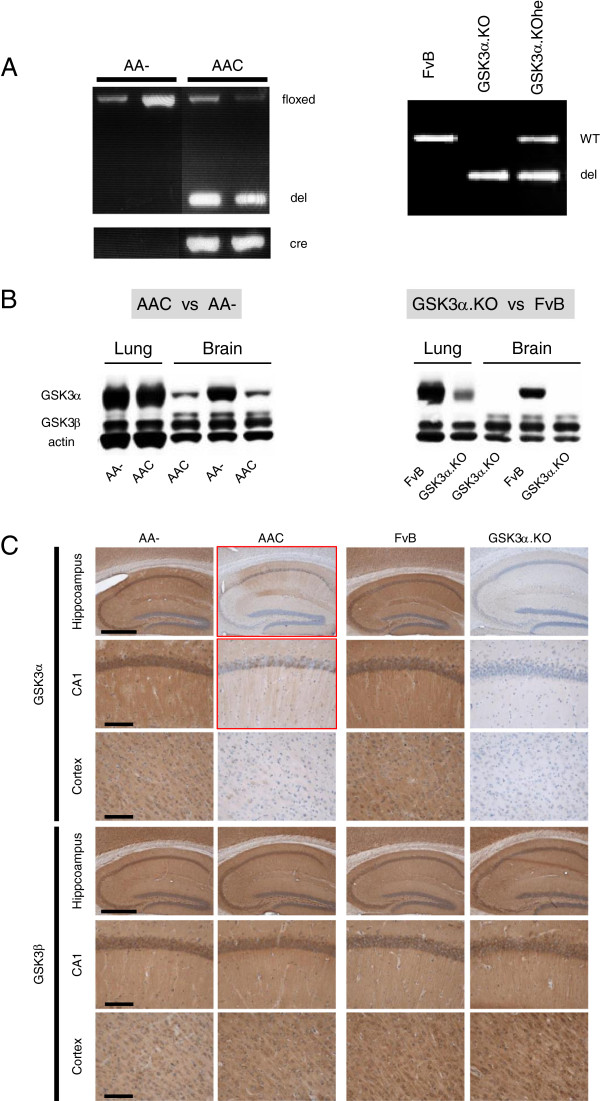
**Genotyping and characterization of neuron-specific and total GSK3α deficient mice. A**. Genotyping by PCR reveals floxed or recombined (del) GSK3α alleles in AAC and AA- mice by the action of Cre-recombinase (Cre) (left panel). In GSK3α.KO mice (right panel) the same PCR reaction defines wild-type (WT) and recombined GSK3α alleles (del) in homozygous and heterozygous GSK3α.KO mice. **B**. Representative western blots for the GSK3 isozymes in total protein extracts of brain and lung from AA-, AAC, FvB and GSK3α.KO mice, as indicated. Note some residual GSK3α protein in the brain of AAC mice as opposed to the total absence in the GSK3α.KO mice. The minor non-specific reaction observed in extracts of lungs of GSK3α.KO mice was caused by a non-identified protein. **C**. Representative IHC of hippocampus, CA1 and cortex for either GSK3 isozyme on brain sections from both deficient genotypes and their respective control mice: AAC versus AA- mice and GSK3α.KO versus wild-type FvB mice. Note some residual GSK3α immunoreaction in the hippocampus of AAC mice, as opposed to total absence in GSK3α.KO mice (cfr text for details).

Offspring was genotyped by classic PCR and by qPCR on DNA extracted from tail-tip biopsies, to define the presence of three possible versions of the mouse GSK3α gene: wild-type, floxed or recombined (Figure 1A). Their occurrence depended on the presence of the Cre-recombinase transgene, established by specific PCR (Figure 1A) [31]. The resulting GSK3α-deficient mice were denoted AAC to indicate the presence of the two floxed recombined GSK3α alleles and of the Cre-recombinase transgene necessary to disrupt the floxed GSK3α genes in neurons. The matching control mice, used throughout the current studies, were denoted AA- because they were homozygous for the floxed GSK3α alleles, but lacked the Cre-recombinase transgene (Figure 1A).

Serendipitously, during the expansion of the AAC colony, we encountered a male pup with a genotype indicative of a homozygous total knock-out of GSK3α: only recombined GSK3α alleles were detected by PCR but not the Cre-recombinase transgene. Offspring of this male founder, sired with female wild-type FvB mice, yielded littermates containing various admixtures of recombined and wild-type GSK3α alleles, as expected for normal Mendelian transmission. Continued breeding yielded offspring that consistently carried only the recombined GSK3α alleles without the Cre-recombinase, confirming that we had produced a mouse strain that lacked active GSK3α genes completely (Figure 1A). We hereby confirmed independently, the viability of mice with total GSK3α deficiency [21]. We denote the totally deficient mice as GSK3α.KO, to differentiate them from the neuron-specific GSK3α deficient AAC mice, described above.

The biological cause of the observed total knock-out, is attributed to the incorporation of mRNA coding for the Cre-recombinase into oocytes, but without the actual genomic transgene itself being present. Subsequent fertilization by AA- sperm can result in germ-line recombination of both maternal and paternal floxed GSK3α alleles, early in the developing fetus. Germ-line transmission of the recombined GSK3α alleles by normal Mendelian inheritance is then no longer dependent of the presence of the Thy1-Cre-recombinase transgene. A similar situation was encountered in the study of the Presenilin gene, although the outcome then was embryonal lethality [31].

Biochemical analysis by western blotting confirmed the strongly decreased levels of GSK3α in brain extracts of AAC mice, relative to the normal levels in the AA- control mice (Figure 1B). In the brain of AAC mice various types of non-neuronal cells maintain GSK3α expression, e.g. glia, endothelial cells and pericytes, because they do not express the Cre-recombinase controlled by the modified mouse Thy1-gene promoter [31]. Consequently, some GSK3α protein was still biochemically detectable in protein extracts of brain of AAC mice, as opposed to the complete absence of GSK3α protein in brain extracts of GSK3α.KO mice (Figure 1B). Western blotting further confirmed GSK3α protein deficiency in lungs (Figure 1B) and in other organs of GSK3α.KO mice.

Immunohistochemistry (IHC) for GSK3α established the strongly decreased reaction in brain sections of AAC mice, and the annihilated staining in GSK3α.KO mice (Figure 1C). Certain types of neurons and their processes maintained weak expression of GSK3α in the AAC mice (Figure 1C; two panels with red borders). Processes and synapses in the stratum lacunosum moleculare (SLM) of AAC mice also retained GSK3α immunoreactivity, although considerably less than in control AA- and wild-type FvB mice (Figure 1C). Of note, the SLM contains synapses of myelinated axons, bundled in the temporoammonic pathway that originates in the entorhinal cortex and projects onto dendrites of CA1 pyramidal neurons. These strata in the CA1 region remain to be explored in detail, because they are proposed to be affected early in AD by “dendritic amputation” [32]. Moreover, the potential contribution of the temporoammonic and perforant pathways to the spreading of pathology in the brain of AD patients is currently subject of considerable interest and debate [33,34].

### GSK3α deficiency did not reflect on GSK3β levels

**Figure 2:**
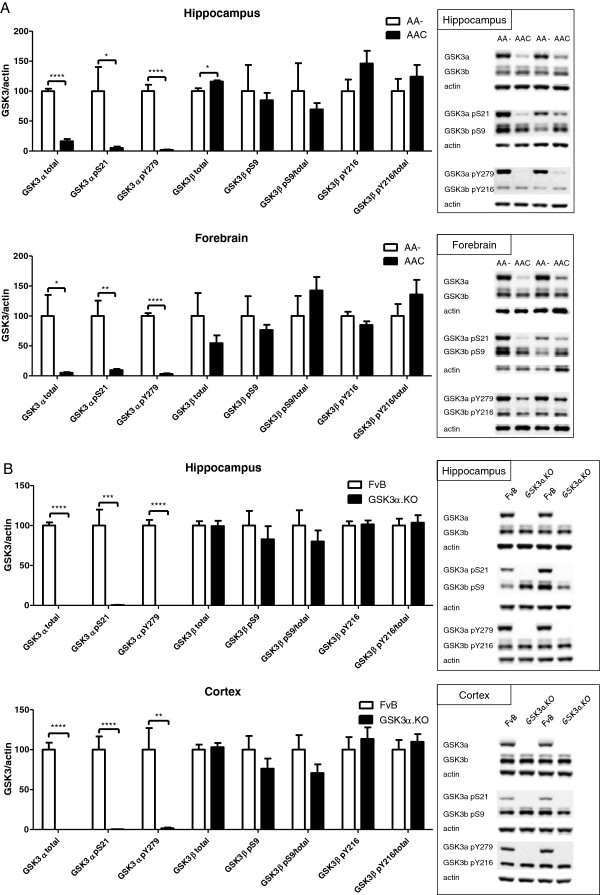
**Biochemical analysis of GSK3 isozymes in mouse brain extracts.** Levels of total GSK3 protein, pS21/S9 and pY279/Y216 in total protein extracts from hippocampus and forebrain from AAC mice compared to control AA- mice (**A**) and from hippocampus and cortex from GSK3α.KO mice versus wild-type FvB mice (**B**). Western blots were digitally quantified, normalized for actin and reported relative to the respective control mice. Data (mean±SEM) are statistically analyzed by unpaired Student’s t-test (two-tailed), n=6 or 7 per genotype; *p<0.05, ** p<0.01, *** p<0.001, **** p<0.0001.

The IHC images and the biochemical data described in the previous section, demonstrated the restricted and complete absence of the GSK3α protein in the newly generated AAC and GSK3α.KO mouse strains, respectively. The biochemical analysis for both GSK3 isoforms furthermore revealed that the GSK3β isozyme was hardly affected by neuronal or by total lack of GSK3α (Figure 1B, Figure 2). We previously concluded for the inverse situation, i.e. in mice with neuron-specific deficiency of GSK3β that expression of GSK3α was hardly affected [23].

That conclusion was now biochemically detailed by analyzing the repercussions of GSK3α deficiency, either neuronal or total, on brain GSK3β. Total protein levels of GSK3β were essentially not affected in the forebrain, nor in the hippocampus or cortex when analyzed separately (Figure 1B, Figure 2A, B). Moreover, the functionally important phosphorylation of GSK3β at pS9 and pY216, were essentially also not affected in the brain of AAC and GSK3α.KO mice, compared to the respective control mice (Figure 2A, B).

The combined observations demonstrated that GSK3α and GSK3β did not effectively compensate for each other in three independent mouse strains. This outcome was in line with the fact that mice lacking GSK3β died around mid-term, obviously not rescued by the remaining normal GSK3α levels [20]. In contrast to the total GSK3β deficiency, the complete lack of GSK3α in mice did not significantly alter their viability at birth, nor their development into adulthood [21], confirmed here independently.

### General phenotypic characteristics of GSK3α-deficient mice

This section presents selected characteristics of both strains of GSK3α-deficient mice to highlight similarities but also marked differences between neuron-specific and total GSK3α deficient mice. Our main interest in neuronal functions led us to analyze in first instance the AAC mice with neuron-specific post-natal deficiency of GSK3α, and compared specified aspects with the neuron-specific GSK3β-deficient mice that we generated recently [23]. In addition, to save valuable time and resources, we took advantage of the GSK3α.KO mice and crossed them with our Tau.P301L mice, to define if GSK3α acted as Tau-kinase in vivo (cfr rationale and data in subsequent sections).

**Figure 3:**
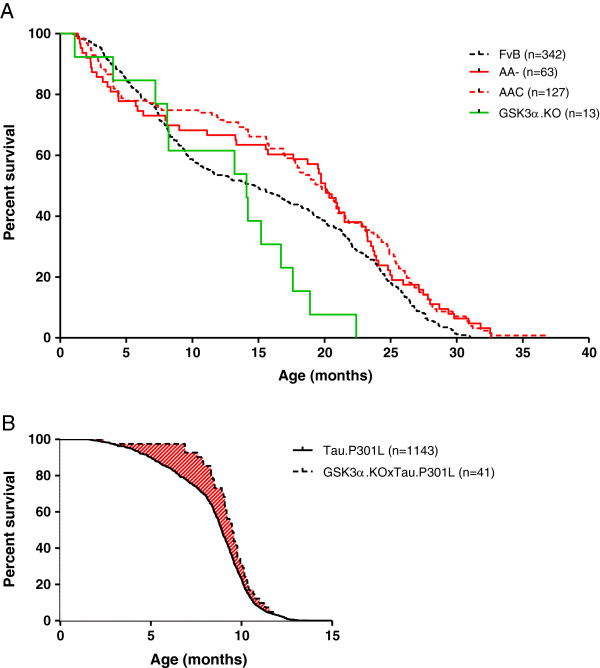
**Kaplan-Meier survival curves.** Spontaneous death of AAC, AA-, GSK3α.KO and wild-type FvB mice (panel **A**) and in GSK3α.KOxTau.P301L bigenic mice versus the parental Tau.P301L mice (panel **B**) housed in the same conditions in our breeding colony. The number of mice of either gender is indicated. Note that earlier death of GSK3α.KO mice (panel **A**) is based on the relatively low number of mice that died spontaneously over the period of observation. The shaded area in panel **B** emphasizes the delayed mortality of young GSK3α.KOxTau.P301L mice, relative to the parental Tau.P301L mice.

The absence of neuronal GSK3α did not reduce the life-expectancy of AAC mice compared to AA- mice (Figure 3A). Although the observed number of GSK3α.KO mice that died spontaneously is low, their lifespan appeared somewhat shorter than that of wild-type mice. Conversely, bigenic GSK3α.KOxTau.P301L mice (detailed below) did not survive longer than the parental Tau.P301L mice, notwithstanding an initial delay in precocious mortality (Figure 3B, arched area) [29,35]. Terminal Tau.P301L mice present with a rapid progression (2–3 weeks) of motor defects and loss in body-weight, upper-airway dysfunction with asphyxia and exhaustion, terminating in precocious death between age 8 and 11 months (mean 9.5 mo) [29,35-37]. The current data imply that GSK3α did not affect this terminal phase, but contributed negatively to the early phenotype of Tau.P301L mice, characterized by defective cognition and beginning motor problems [29,35,36].

**Figure 4:**
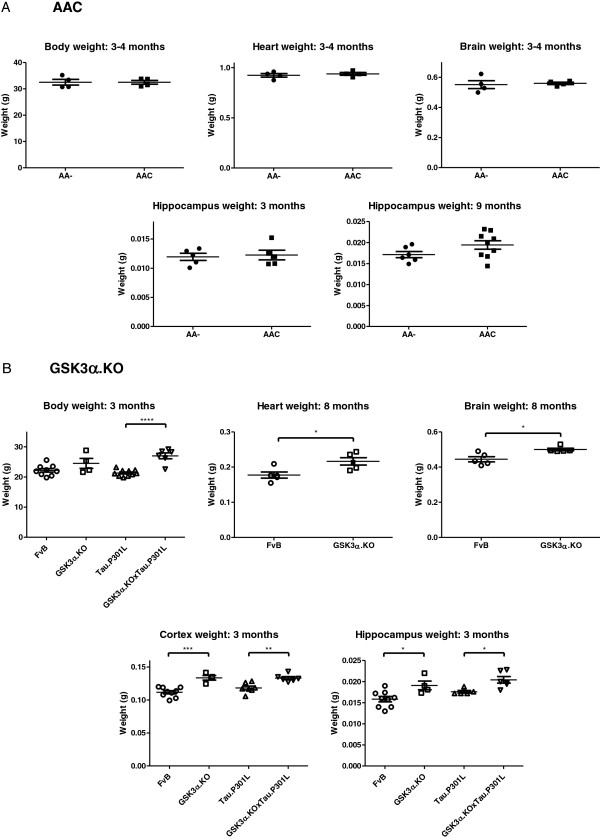
**Body weight and brain weight.** Body weight, wet weight of heart, brain, hippocampus of (**A**) AAC and AA- mice of either gender at young (n=4/5) and older age (n=6/9) and (**B**) of GSK3α.KO, GSK3α.KOxTau.P301L, Tau.P301L and FvB mice (n=4/10) at young and older age. Data (mean±SEM) are statistically analyzed by unpaired Student’s t-test (two-tailed), * p<0.01, ** p<0.05, *** p<0.001, **** p<0.0001.

The average body-weight of AAC and AA- mice was comparable (Figure 4A), while GSK3α.KO mice were on average heavier than age- and gender-matched wild-type mice (Figure 4B). We thereby confirmed and extended data of a similar strain of mice with total GSK3α deficiency, generated and characterized independently [21]. Conversely, neuron-specific GSK3α deficiency did not reflect on body-weight, implicating that GSK3α affected body-weight not by central actions, but by mechanisms operating in peripheral organs and systems.

**Figure 5:**
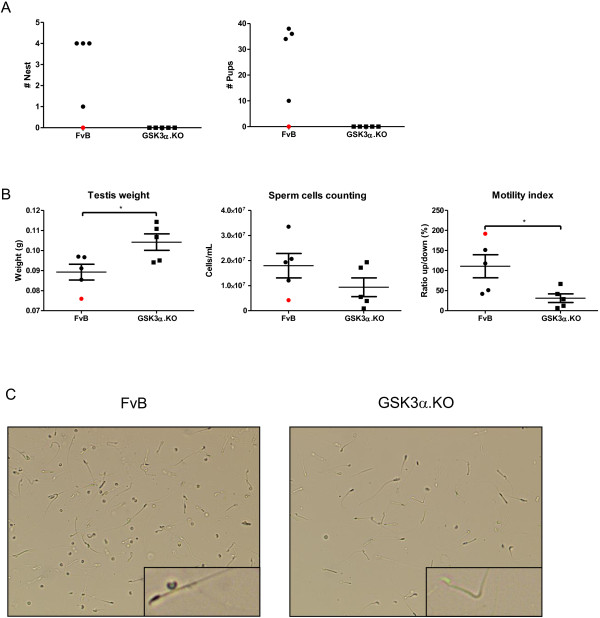
**Male GSK3α.KO mice are infertile. A**. Breeding performance of GSK3α.KO mice versus FvB mice, estimated as number of pups and number of litters over a period of 111 days from 5 breeding couples/genotype). **B**. Testis weight, sperm cell counting and motility index assessed in males (n=5). All data (mean±SEM) are statistically analyzed by unpaired Student’s t-test (two-tailed): Testis weight, * p=0.030; sperm cells, p=0.198; motility index, * p=0.032. The red symbols in panels **A** and **B** represent a sterile FvB male. **C**. Sperm cells from GSKα.KO and FvB wild-type mice. Inset: single sperm at higher magnification.

The neuron-specific deficiency of GSK3α did not modify the gross neuro-anatomical features of AAC mice (Figure 1C, Figure 4A), consistent with the similar wet weight of total brain and of hippocampus, both at young (3–4 months) and advanced age (9 months) (Figure 4A). In contrast, the hippocampus and cortex were significantly heavier in adult female GSK3α.KO mice than in wild-type FvB females (Student’s t-test, p=0.0004 and p=0.0181 respectively, Figure 4B). Adult male GSK3α.KO mice presented with a significant heavier brain (Student’s t-test, p=0.0212, Figure 4B), in line with their overall more sturdy anatomy, including a larger heart and testis than wild-type males (Figure 4B, Figure 5). Our data confirmed that the size of brain and peripheral organs were differently affected by the absence of GSK3α [21].

Surprisingly, homozygous GSK3α.KO male mice proved to be infertile, in contrast to wild-type FvB mice that only very rarely produced a sterile male: we observed one individual in this study (represented by red symbol in Figure 5A). The AAC male mice sired offspring normally, with the number of litters and the number of pups per litter very similar to the control AA- males (Figure 5A). While the testes of male GSK3α.KO mice were significantly larger than those of wild-type male mice, they produced similar, or somewhat lesser numbers of sperm cells (Figure 5B). Conversely, the motility of spermatozoids collected from the cauda epididymis of homozygous GSK3α.KO male mice, assessed by the swim-up test, was significantly defective (31.1% vs 110.8% in male wild-type mice; Student’s t-test, p=0.032) (Figure 5B). Moreover, spermatozoids produced by homozygous GSK3α.KO males were morphologically abnormal, presenting with kinky flagellae, while sperm heads appeared morphologically normal (Figure 5C). We concluded that the total deficiency of GSK3α profoundly disturbed the morphology and the motility of sperm, causing infertility of GSK3α-deficient males, in contrast to normal fertility of neuron-specific GSK3α-deficient male mice.

### Neurological characteristics of mice with neuron-specific GSK3α deficiency

#### Morphology of CA1

**Figure 6:**
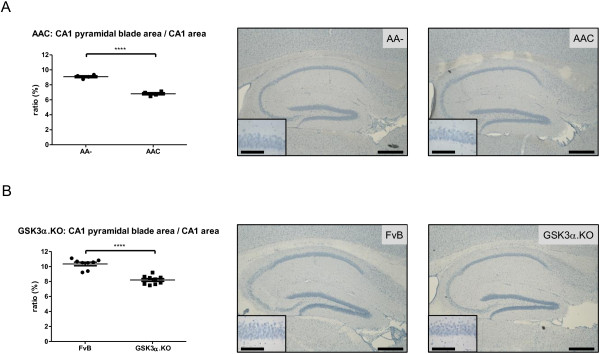
**Reduced CA1 in neuron-specific and in total GSK3α-deficient mice.** Left panels: ratio of CA1 pyramidal neurons to total CA1 area in (**A**) AA- and AAC mice (n=4; 3 sections/mouse) and (**B**) FvB and GSK3α.KO mice (n=8/9; 3 sections/mouse). Data (mean±SEM) are statistically analyzed by unpaired Student’s t-test (two-tailed), **** p<0.0001. Right panels: representative images of hippocampus and CA1 pyramidal layer (insets). Scale bars 400 μm; insets: 100 μm.

The CA1 pyramidal brain layer in AAC mice was shorter and thinner than in control AA- mice (Figure 6A), whereas the overall wet weight of the hippocampus did not differ (Figure 4A). The surface of the CA1 pyramidal blade relative to the total CA1 area, was significantly reduced in AAC mice compared to AA- mice (respectively 6.81% vs. 9.09% p<0.001) (Figure 6A). The significant reduction of the CA1 pyramidal blade relative to the total CA1 area was also evident in GSK3α.KO mice compared to wild-type mice (8.20% vs 10.35% p<0.001) (Figure 6B) despite the increased hippocampal wet weight (Figure 4B). Both sets of data consolidated the conclusion that besides GSK3β, also GSK3α contributes to the neuronal architecture of the hippocampus, and interestingly, to the CA1 sub-region that is most vulnerable in AD.

#### Electrophysiology & LTP

**Figure 7:**
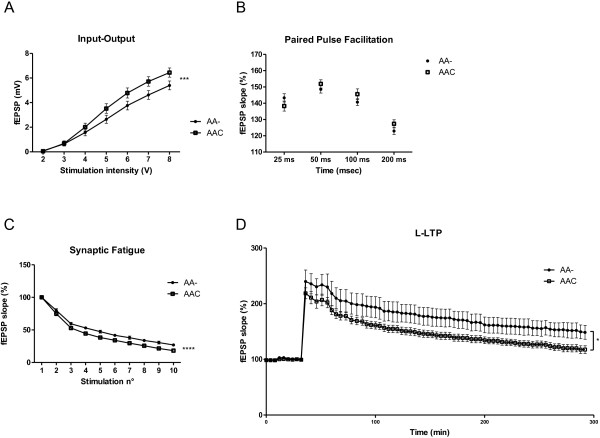
**Electrophysiology of neuron-specific GSK3α deficient mice. A**. Input–output curve generated by gradually increasing stimulus intensity in sections from AAC mice compared to control AA- mice (n=6). Two-way Anova; genotype: F_(1,399)_=15.20, *** p<0.0001; stimulation intensity: F_(6,399)_=106.9, p<0.0001; interaction: F_(6,399)_=1.256, p=0.2766. **B**. Paired pulse facilitation (PPF) induced in 4 intervals of 25, 50, 100 and 200 ms in CA1 stratum radiatum of sections from AAC mice compared to AA- mice (n=6). Data are expressed as the ratio of the second to the first fEPSP slope. Two-way Anova; genotype: F_(1,220)_=1.124, p=0.2903; time: F_(3,220)_=34.35, p<0.0001; interaction: F_(3,220)_=1.685, p=0.1710. **C**. Synaptic fatigue analyzed for 1 to 10 stimulations in sections from AAC mice compared to AA- mice (n=6). Two-way Anova; genotype: F_(1,320)_=67.23, **** p<0.0001; stimulus: F_(9,320)_=321.3, p<0.0001; Interaction: F_(9,320)_=1.030, p=0.4153. **D**. LTP was recorded in CA1 stratum radiatum in sections from AAC and AA- mice (n=6). L-LTP was analyzed during the last 2 hours of recording. Unpaired Student’s t-test (two-tailed); *p=0.0369.

The repercussions of lacking GSK3α activity at the synaptic level was assessed electrophysiologically in acute hippocampal sections from AA- and AAC mice. We measured classic LTP as well as pre- and post-synaptic parameters in CA1 stratum radiatum (SR) [14]. The recorded input–output characteristic was significantly higher in AAC mice (p<0.0001; Figure 7A) pointing to important alterations in basal synaptic transmission imposed by the missing GSK3α activity. Conversely, paired pulse facilitation (PPF) was similar in sections from AAC and AA- mice (Figure 7B) while synaptic fatigue was significantly reduced in AAC mice (p<0.0001; Figure 7C). The data implied that GSK3α controlled important pre-synaptic physiological mechanisms, and that these functions were not compensated for, nor taken over by GSK3β.

Late-LTP (L-LTP), recorded up to 4.5 hours post-stimulus, was significantly reduced in AAC mice relative to AA- mice (during the last 2 hours p=0.0369; Figure 7D). The finding argued for a post-synaptic defect caused by the absence of GSK3α, and implied a physiological function for GSK3α in post-synaptic signal handling, in addition to the accepted contribution of GSK3β [5,7,14,16,38].

The combined electrophysiological data demonstrated, somewhat unexpectedly, that synaptic transmission was importantly deregulated by pre- and post-synaptic actions in mice with neuron-specific deficiency of GSK3α. These findings instigated us to characterize in more detail the motor capacity, the general behavior and the cognition of the neuron-specific GSK3α-deficient mice.

### Motor activity and behavior

AAC mice were subjected to classical motor tests but no important motor-related impairments were noted (Table 1). Moreover, the AAC mice never showed clasping of the legs, not even at advanced age (18 months), which combined with the motor tests demonstrated that GSK3α did not contribute appreciably to motor-neuron activity.

**Table 1:** Motor ability, cognition and pain sensitivity of AA- and AAC mice (age 3–4 mo)

**Task**	**Parameter (units)**	**AA-**	**AAC**	**p**
**Motor**				
Inverted grid	Time (sec)	60.00±0.00	58.40±1.60	N/A
Beamwalk	Time (sec)	180.00±0.00	171.80±8.25	N/A
**Behavior & cognition**				
Y-maze	Index	75.06±2.59	69.42±2.69	0.1475
	Entries (n)	27.91±2.55	26.75±1.54	0.6956
	Alterations (n)	19.55±2.11	17.08±1.12	0.3031
OFT	Corners (sec)	338.60±24.53	312.30±23.31	0.4469
	Periphery (sec)	556.70±8.67	561.70±5.89	0.6331
	Center (sec)	43.36±8.67	38.36±5.89	0.6331
	Distance moved (cm)	3972±444.4	4327±397.0	0.5573
	Mobility (%)	66.77±4.02	68.75±4.23	0.7385
Dark/Light	Latency (sec)	52.27±24.46	55.17±24.84	0.9348
	Transitions (n)	4.18±0.74	4.33±0.75	0.8872
	Time spent in light (sec)	106.60±28.04	89.00±15.24	0.5776
Porsolt swim test	Immobility (%)	53.96±4.78	49.55±3.70	0.4673
* NORT	Index	59.56±3.78	45.82±3.76	0.0176
	Distance moved (cm)	2158±293.4	2040±273.4	0.7712
	Mobility (%)	37.05±4.04	33.78±3.96	0.5708
PIA	Latency to dark (sec)	180.50±36.35	248.60±25.77	0.1334
**Pain sensitivity**				
Footshock threshold	Flinching (mA)	0.1350±0.0183	0.1792±0.0208	0.1347
	Jumping (mA)	0.4833±0.0546	0.6042±0.0711	0.2189
	Vocalizations (mA)	0.3300±0.0300	0.3875±0.0418	0.2948
* Hot plate task	Time (sec)	8.90±0.74	6.92±0.43	0.0256
* Capsaicin sensitivity	Wipes (n)	60.33±5.82	82.91±5.75	0.0136

Abbreviations: *OFT*, open-field test; *NORT*, novel object recognition task; *PIA*, passive inhibitory avoidance; *, denote statistically significant differences.

AAC mice displayed unaltered anxiety-related behavior, assessed in the classical open-field test (OFT). Both AAC and AA- mice spent similar fractions of the allotted test-time in the corners, the center and the periphery of the arena (Table 1). Moreover, the distance travelled by mice from either genotype was very similar, and so was their overall total activity in the open-field, again underwriting the conclusion that GSK3α deficiency did not cause motor problems in the AAC mice (Table 1).

The conclusion that neuronal deficit of GSK3α affected neither motor activity nor anxiety related behavior, was further confirmed in the dark–light paradigm. This well-known task was developed to estimate anxiety, and has been used to measure the effect of anxiolytic and anxiogenic drugs [39,40]. Neither the latency to enter the lighted compartment, nor the time spent there, nor the number of transitions between dark and lighted compartments differed markedly between the AAC and AA- mice (Table 1).

Depressive or depression-like behavior was assessed by the Porsolt forced-swim task, a robust well-established paradigm in rodents [41,42]. Mice were forced to swim in a small water-tank from which they could not escape, and after an initial bout of forced swimming the mice assumed an immobile floating position. The relative time of passive floating (immobility) was taken as measure of behavioral despair, observed to be decreased by anti-depressants [41]. The test did, however, not discriminate AAC and AA- mice (Table 1), indicating that AAC mice were not depressed or in a depression-like condition.

The combined data demonstrated that neuronal GSK3α deficiency did not appreciably impinge on motor activity or on general behavior, nor induced anxiety in the AAC mice.

### Cognition: Y-maze & NORT

The morphological and electrophysiological defects of the hippocampal region, described above, led us to analyze whether GSK3α was involved in cognitive processes, besides GSK3β. We assessed the cognitive performance of the AAC mice relative to control AA- mice in the Y-maze to measure their working memory [43,44], and in the novel object recognition task to assess hippocampus-dependent learning and memory [31,45,46].

In the Y-maze, AAC mice did not show differences in either the number of entries or alternations and displayed only a trend towards a decrease in spontaneous alternations (Table 1).

In the novel object recognition task (NORT), imposed 4 hours following suitable training, the AAC mice exhibited a significant decreased recognition index compared to AA- mice (p=0.0176; Table 1), without a difference in distance traveled or mobility (Table 1). The test was repeated and the outcome confirmed in three different cohorts of age- and gender-matched AAC and AA- mice. The compelling evidence for a distinct memory impairment of the AAC mice, thereby established a function of GSK3α in synaptic plasticity, underpinning our electrophysiological data.

### Cognition: PIA

We compared AAC & AA- mice in the cognitive task of passive inhibitory avoidance (PIA), presented here separately for several reasons. On the one hand, PIA surprisingly revealed a trend, not for defective but for improved cognition of the AAC mice (Table 1). On the other hand, PIA revealed a markedly increased sensitivity of the AAC mice to the electric footshocks they received during their conditioning for the PIA task. These unexpected observations led us to analyze the pain-sensitivity of the AAC mice.

The reaction to an electric foot-shock was in itself not exacerbated by the neuron-specific GSK3α deficiency: the AAC mice showed similar intensity thresholds as the control AA- mice for jumping, vocalization and flinching in response to foot-shocks (Table 1). In contrast, thermal nociception measured by the hotplate test (55°C) revealed a significantly lower latency for paw-withdrawal in the AAC mice (p=0.0256; Table 1).

We analyzed the potential implication of the capsaicin receptor TRPV1 in these sensory reactions, by the capsaicin eye sensitivity test [47]. The number of wiping actions with the forelimbs following administration of a diluted capsaicin solution to the eyes, was significantly higher in the AAC mice than in the AA- mice (p=0.0136; Table 1). The TRPV1 channel, well-known to control nociception was therefore analyzed in trigeminal ganglia and dorsal root ganglia from AAC and AA- mice by q-RT-PCR. No differences in TRPV1 mRNA levels were observed, while also brain TRPV1 protein levels, analyzed by western blotting were not affected in the AAC mice. We consequently excluded a TRPV1-mediated mechanism in the augmented pain sensitivity invoked by neuron-specific GSK3α deficiency.

### Biochemical modification of GSK3 isozymes by cognitive training of wild-type mice

Biochemical analysis of the brain of AAC mice surprisingly demonstrated that performing the PIA task itself led to biochemical modifications of the GSK3 isozymes. To define whether cognitive training was directly responsible, we biochemically analyzed hippocampal protein extracts from wild-type mice following cognitive training in PIA and NORT for total GSK3α/β levels, and for phosphorylated pS21/S9 and pY279/Y216 isoforms.

**Figure 8:**
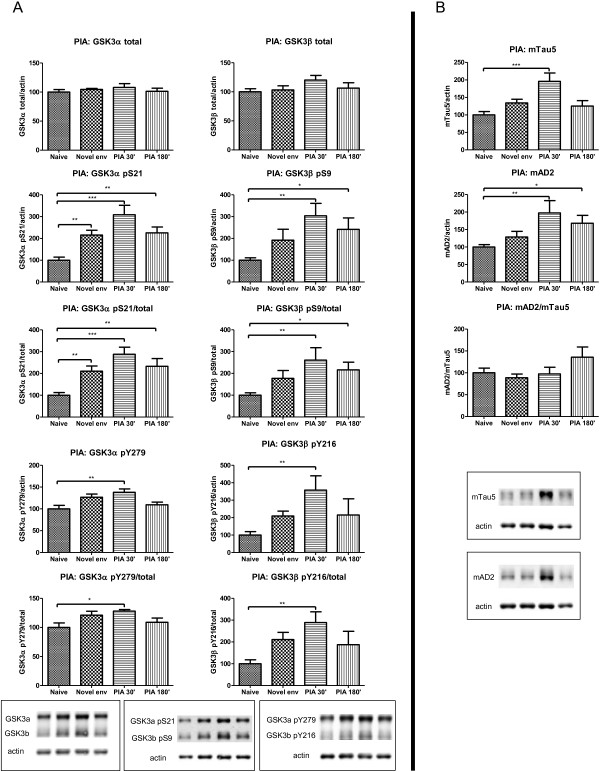
**Biochemical repercussions of PIA on GSK3 and protein tau in hippocampus of wild-type mice. A**. Biochemical analysis of GSK3 by western blotting of total protein extracts from hippocampus of FvB wild-type mice (n=10) at different timepoints before or after they performed the PIA task (n=6 per timepoint). All data normalized to actin and reported relative to naive control mice. Data (mean±SEM) are statistically analyzed by one-way Anova (Dunnet’s *post hoc* test compared to naive). GSK3α: F_(3,24)_=0.5422; p=0.6581. GSK3β: F_(3,24)_=1.523; p=0.2339. GSK3α pS21: F_(3,24)_=12.58; p<0.0001. GSK3α pS21/total: F_(3,24)_=12.11; p<0.0001. GSK3β pS9: F_(3,24)_=5.240; p=0.0063. GSK3β pS9/total: F_(3,24)_=4.832; p=0.0090. GSK3α pY279: F_(3,24)_=4.973; p=0.0080. GSK3α pY279/total: F_(3,24)_=3.346; p=0.0359. GSK3β pY216: F_(3,24)_=3.875; p=0.0216. GSK3β pY216/total: F_(3,24)_=4.528; p=0.0119. * p<0.05; ** p<0.01; *** p<0.001. Lower panels show representative western blots. **B**. Biochemical analysis of protein Tau by western blotting of total protein extracts from hippocampus of FvB wild-type mice (n=10) at different timepoints before or after they performed the PIA task (n=6 per timepoint). All data normalized to actin and reported relative to naive control mice. Data (mean±SEM) are statistically analyzed by one-way Anova (Dunnet’s *post hoc* test compared to naive). Tau5: F_(3,24)_=7.625; p=0.0009. AD2: F_(3,24)_=5.162; p=0.0068. AD2/Tau5: F_(3,24)_=1.761; p=0.1815. * p<0.05; ** p<0.01; *** p<0.001. Lower panels show representative western blots.

**Figure 9:**
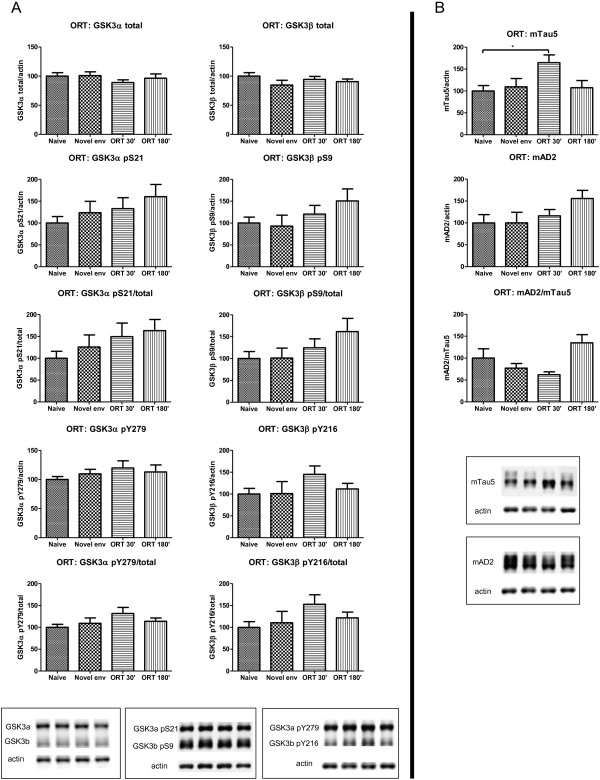
**Biochemical repercussions of NORT on GSK3 and Tau in hippocampus of wild-type mice. A**. Biochemical analysis of GSK3 by western blotting of total protein extracts from hippocampus of FvB wild-type mice (n=10) at difference timepoints before or after they performed the NORT task (n=6 per timepoint). All data normalized to actin and reported relative to naive control mice. Data (mean±SEM) are statistically analyzed by one-way Anova (Dunnet’s *post hoc* test compared to naive). GSK3α: F_(3,24)_= 0.6609; p=0.5842. GSK3β: F_(3,24)_= 1.131; p=0.3566. GSK3α pS21: F_(3,24)_= 1.351; p=0.2816. GSK3α pS21/total: F_(3,24)_= 1.529; p=0.2327. GSK3β pS9: F_(3,24)_= 1.469; p=0.2479. GSK3β pS9/total: F_(3,24)_= 1.699; p=0.1939. GSK3α pY279: F_(3,24)_= 1.002; p=0.4091. GSK3α pY279/total: F_(3,24)_= 1.871; p=0.1614. GSK3β pY216: F_(3,24)_= 1.302; p=0.2967. GSK3β pY216/total: F_(3,24)_= 1.580; p=0.2203. Lower panels show representative western blots. **B**. Biochemical analysis of protein Tau by western blotting of total protein extracts from hippocampus of FvB wild-type mice (n=10) at difference timepoints before or after they performed the NORT task (n=6 per timepoint). All data normalized to actin and reported relative to naive control mice. Data (mean±SEM) are statistically analyzed by one-way Anova (Dunnet’s *post hoc* test compared to naive). Tau5: F_(3,24)_= 3.286; p=0.0380. AD2: F_(3,24)_= 1.637; p=0.2071. AD2/Tau5: F_(3,24)_= 2.593; p=0.0761. * p<0.05. Lower panels show representative western blots.

Total GSK3α/β protein levels were not affected by the PIA task, but the inhibitory phosphorylation at S21/S9 in GSK3α/β were significantly increased (Figure 8A). The same trend of inhibitory serine phosphorylation of both GSK3 kinases was observed in wild-type mice subjected to NORT (Figure 9A). Concomitant with these indications for decreased kinase activity of both GSK3 isozymes, a moderate increase in phosphorylation of Y279/Y216 in GSK3α/β was evident when brain was analyzed 30 min after the PIA-test.

Surprisingly, the foregoing biochemical observations extended to protein Tau in the same brain extracts: total levels of endogenous mouse Tau were increased in the hippocampus of wild-type mice subjected to the PIA and NORT tasks (Figure 8B; Figure 9B). The possible mechanisms underlying these observations, and their eventual relation, remain unknown and deserve to be explored, because they comply with recent observations that lower tau levels profoundly affect physiological and pathological neurological characteristics [48-52].

### Comparative analysis of GSK3α.KO mice and GSK3α.KOxTau.P301L bigenic mice

While GSK3β proved to be a very effective Tau-kinase in mouse brain, with important functional consequences [26-29,36], GSK3α remained largely overlooked in this respect. Here we investigated the contribution of GSK3α to the phosphorylation of protein Tau in the GSK3α.KOxTau.P301L bigenic mice that we derived specifically for this goal. The alternative, i.e. to generate AACxTau.P301L bigenic mice, would have required a much more complex genetic assembly, because 5 alleles would have been needed: (i) homozygosity of the floxed GSK3α alleles, (ii) homozygosity of the transgenic Tau.P301L alleles, (iii) at least one allele of the Thy1 Cre-recombinase transgene. To save valuable time and effort we decided to restrict our investigation to the GSK3α.KOxTau.P301L bigenic mice, which were more straightforward to breed, and yielded the same information.

### Motor behavior

We compared GSK3α.KO and bigenic GSK3α.KOxTau.P301L mice by most of the behavioral and cognitive tests also performed with the AAC mice, described in foregoing sections. Both genotypes were compared to their appropriate age- and gender-matched controls, c.q. GSK3α.KO to wild-type FvB mice and GSK3α.KOxTau.P301L to the parental Tau.P301L mice.

The GSK3α.KO mice did not show clasping of the legs, nor any impairment on the rotarod (Table 2), which extended our observations on the AAC mice and strengthening the conclusion that GSK3α did not importantly affect motor ability.

**Table 2:** Motor ability and cognition of young female GSK3α.KO and GSK3α.KOxTau.P301L mice

**Task**	**Parameter**	**FvB**	**GSK3α.KO**	**p**	**Tau.P301L**	**GSK3α.KO x Tau.P301L**	**p**
			**Motor**				
* Rotarod	Time (sec)	299.4±0.6	300.0±0.0	NA	293.3±5.5	254.6±17.4	0.0268
			**Behavior & cognition**				
* Y-maze	Index	66.95±1.66	71.15±4.84	0.3553	69.91±3.46	62.80 ±5.28	0.2600
*	Entries (n)	42.22±2.84	23.17±2.06	0.0003	45.10±1.49	27.38±3.18	<0.0001
*	Alterations (n)	26.89±1.88	15.00±1.67	0.0007	30.10±1.74	16.13±2.16	0.0001
* OFT	Corners (sec)	292.7±11.6	380.0±28.9	0.0070	282.3±11.10	337.8±29.6	0.0745
	Periphery (sec)	549.9±8.08	572.8±7.08	0.0677	539.0±6.55	554.8±11.09	0.2169
	Center (sec)	50.15±8.08	27.19±7.08	0.0677	61.02±6.55	45.22±11.09	0.2169
*	Distance (cm)	5972±303.7	3315± 389.6	0.0001	6500±341.3	3178±442.2	<0.0001
*	Mobility (%)	74.12±1.41	53.00±4.59	0.0002	77.72±1.09	55.08±5.95	0.0007
* NORT	Index	77.21±4.61	69.71±1.25	0.2189	78.46±1.90	59.91±7.14	0.0075
*	Distance (cm)	4017±263.5	2319± 329.9	0.0014	4459±303.6	1924±411.6	0.0002
*	Mobility (%)	66.78±1.96	44.86±5.09	0.0005	71.49±2.03	41.91±7.88	0.0005
* Dark/Light	Latency (sec)	21.67±4.90	27.33±3.99	0.4242	19.20±4.25	11.88±2.97	0.1985
*	Transitions (n)	11.11±1.01	6.17±1.05	0.0059	11.00±0.54	4.625±0.80	<0.0001
*	Time in light (sec)	150.40±4.49	132.5±29.08	0.4669	139.30±6.51	74.88±17.52	0.0018

Abbreviations: *OFT*, open-field test; NORT, novel object recognition task; PIA, passive inhibitory avoidance; * denote statistically significant differences.

It was therefore surprising to note that the bigenic GSK3α.KOxTau.P301L mice performed significantly worse than Tau.P301L mice on the rotarod task, already at young age (1–2 months) (p=0.0268; Table 2). We consistently observed and reported that the parental Tau.P301L mice became motor impaired only at adult age (6–8 months). These defects correlated with the progressive phosphorylation of protein Tau.P301L, and were attributed to the sensitivity of motor neurons to beginning tauopathy [29,35,36]. Obviously, the responsible mechanisms must be more complex because the lack of GSK3α was not expected to increase, but rather to decrease the phosphorylation of protein Tau, as experimentally observed (cfr next section).

### Behavior and cognition

In the open-field test, young GSK3α.KO mice tended to spend more time in the corners and less in the center of the arena compared to matched wild-type mice (p=0.0070 and p=0.0677 respectively; Table 2). The distance moved, as well as the speed and total activity were all lowered by GSK3α deficiency. Because motor problems were not indicated by the clasping and rotarod tests, we concluded stress-induced anxiety to be responsible, possibly reflecting a depression-like state or even mania, as proposed for GSK3 overexpressing mice [53,54]. Very similar behavior of bigenic GSK3α.KOxTau.P301L mice was observed, including significantly decreased travel and mobility in the open field (p<0.0001 and p=0.0007 respectively; Table 2) demonstrating that no additional problems were conferred in the bigenic combination on either of the parental phenotypes.

The cognitive performance, measured in the Y-maze task, revealed similar highly significant defects in GSK3α.KO and GSK3α.KOxTau.P301L mice in the number of entries and in alternations (p=0.0003 and p=0.0007 respectively; Table 2), although the index did not differ between either type of GSK3α-deficient mice and their respective controls. In NORT, the GSK3α.KO and GSK3α.KOxTau.P301L mice presented both with a minor cognitive impairment, and their decreased general activity and locomotion confirmed the open-field observations (Table 2).

In the dark–light paradigm, the GSK3α.KO mice tended towards increased latency to enter the lighted compartment (Table 2) but with a highly significant decrease in the number of transitions (p=0.0059; Table 2). The GSK3α.KOxTau.P301L mice were similarly impaired as the GSK3α.KO mice in the number of transitions (p<0.0001; Table 2) but, in addition, spent significant less time in the lighted sector than age-matched young Tau.P301L mice (p=0.0018; Table 2).

The combined data demonstrated interestingly distinct contributions of GSK3α to cognition and to anxiety-related behavior of mice. This was exacerbated by the pathological contribution of the onset of tauopathy, which were nevertheless still minor in the young GSK3α.KOxTau.P301L mice (age 1–2 months). Conversely, both GSK3α.KO and GSK3α.KOxTau.P301L mice were hypoactive in several tasks, compared to their respective controls, which was not caused by motor deficits.

### GSK3α as Tau kinase in brain in vivo

We first assessed the contribution of GSK3α to the phosphorylation of endogenous mouse protein Tau (mTau) in AAC and GSK3α.KO mice, compared to AA- and wild-type mice, respectively. In second instance, biochemical analysis was extended to human protein Tau.P301L in the bigenic GSK3α.KOxTau.P301L combination compared to the parental Tau.P301L mice.

### Decreased phosphorylation of endogenous mouse Tau in AAC and in GSK3α.KO mice

**Figure 10:**
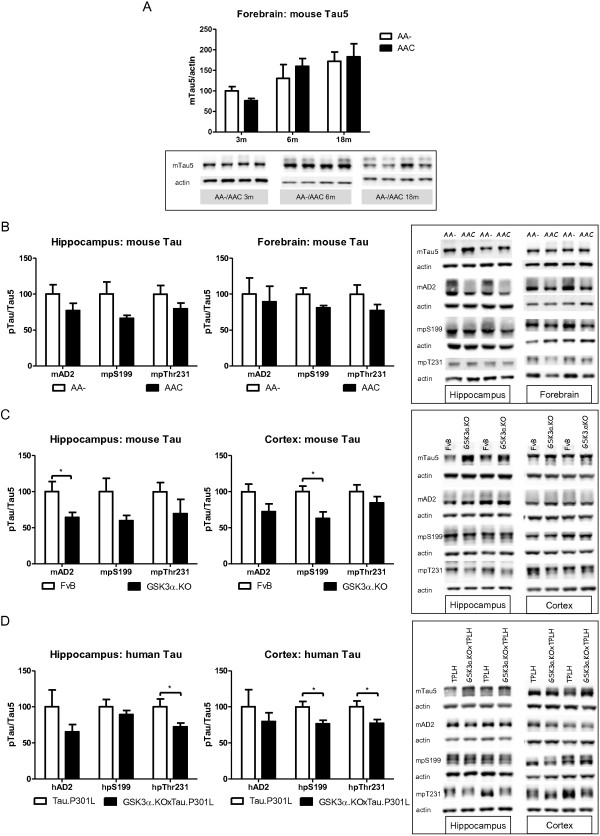
**Biochemical analysis of protein Tau in GSK3α deficient mice. A**. Biochemical analysis by western blotting of total protein extracts from forebrain of AAC and AA- mice aged 3, 6 and 18 months (n=5/age). Tau protein levels are normalized to actin and expressed relative to control AA- mice at age 3 months. Data (mean±SEM) are statistically analyzed by two-way Anova (Bonferroni *post hoc* test), genotype: F_(1,24)_=3.40, p=0.5093; age: F_(2,24)_=37.60, p=0.0027; interaction: F_(2,24)_=0.20, p=0.7759. Lower panels show representative western blots. **B**. Biochemical analysis by western blotting for phospho-epitopes pS396/404, pS199 and pT231 of endogenous mouse Tau in total protein extracts from hippocampus and forebrain of AAC and control AA- mice. **C**. Biochemical analysis by western blotting for phospho-epitopes pS396/404, pS199 and pT231 of endogenous mouse Tau in total protein extracts from hippocampus and cortex of GSK3α.KO and FVB wild-type mice. **D**. Biochemical analysis by western blotting for phospho-epitopes pS396/404, pS199 and pT231 of human Tau.P301L in total protein extracts from hippocampus and cortex of GSK3α.KOxTau.P301L mice and the parental Tau.P301L mice. In panels **B-D**, all data are normalized for total Tau and reported relative to the respective control mice. Data (mean±SEM) are statistically analyzed by unpaired Student’s t-test (two-tailed), n=6 or 7 per genotype; * p<0.05.

We analyzed biochemically total protein extracts of the forebrain of AAC and AA- mice ranging in age from 3 to 18 months. No major differences between AAC and AA- mice were evident in total forebrain endogenous mTau levels, while a progressive increase with age was observed to be similar in both genotypes (Figure 10A).

We went on to analyze three major phosphorylated epitopes of mTau, i.e. pS199, pT231 and pS396/pS404, because these are well-known physiological and pathological markers. They are proposed to depend on GSK3 activity, although to a different extent depending on the test system and model. In hippocampal and forebrain extracts of AAC mice we noted the general trend for decreased phosphorylation of mTau at all three epitopes, indicating a contribution of GSK3α (Figure 10B). In GSK3α.KO mice phosphorylation of mTau was decreased in the hippocampus. Especially the level of AD2 was significantly reduced by the absence of GSK3α, although it is generally presented as a GSK3β dependent epitope (Figure 10C). The reduced phosphorylation of mTau was further sustained by the significantly decreased levels of pS199 in the cortex of GSK3α.KO mice (Figure 10C). Obviously, GSK3α contributed to the physiological phosphorylation of various sites on endogenous protein Tau in mouse brain.

### Decreased phosphorylation of human Tau.P301L in brain of GSK3α.KOxTau.P301L mice

Similar biochemical analysis of human Tau.P301L in the brain of bigenic GSK3α.KOxTau.P301L mice revealed the generalized trend of decreased phosphorylation at the same epitopes analyzed. The level of pT231 was significantly decreased in hippocampus and in cortex of GSK3α.KOxTau.P301L mice compared to Tau.P301L mice, while the pS199 levels were significantly decreased in the cortex (Figure 10D).

Although the three phosphorylated epitopes that we analyzed are classically attributed to depend on the activity of the GSK3β isozyme, our current data demonstrated that also the less well studied GSK3α isozyme contributed to the phosphorylation of protein Tau at these physiologically and pathologically important phospho-epitopes.

## Discussion

To clarify the neurological functions of GSK3α, the isoform that is far less well studied than GSK3β, we generated mice that were deficient in GSK3α, specifically in neurons. Available mice with floxed GSK3α genes [30] were crossed with Thy1-Cre mice, with neuron-specific expression of Cre recombinase, first used for neuronal Presenilin1 gene inactivation [31]. In addition, we took advantage of the fortuitous finding of offspring with total GSK3α deficiency (GSK3α.KO). Both strains were expanded, and moreover GSK3α.KO mice crossed with Tau.P301L mice, yielding unique models that define GSK3α as Tau kinase in vivo.

The most marked observations combined from all GSK3α-deficient mice are discussed: (i) striking independent regulation of the GSK3 isozymes; (ii) numerous neuroanatomical, behavioral and cognitive traits depending on GSK3α; (iii) physiological and pathological phosphorylation of protein Tau.

We finally discuss the regulation of GSK3 activity by phosphorylation during behavioral tasks.

### GSK3 isozymes act independently

Biochemical data, presented here and before [23] demonstrate conclusively that tampering with one of the GSK3 isozymes do not noticeably affect the other. Both GSK3 isozymes, despite their close structural and functional relations, are controlled independently of each other. Physiologically, they share substrates, but have preferred sets of substrates in brain [55]. Translational implications are not evident, i.e. specific inhibitors of either isozyme would not per se be better for treating neurological diseases.

### GSK3α contributes to neuroanatomy, behavior and cognition

In sharp contrast to GSK3β, complete deficiency of GSK3α during mouse embryogenesis allows normal development even including CNS. The marked exception of male spermatogenesis, cannot be discussed here in detail for lack of analytical data, but GSK3α appears more essential than GSK3β for normal development and functioning of sperm-flagellae.

We concentrated specifically on CNS but observed normal brain anatomy in mice with total or with neuron-specific deficiency of GSK3α. The interesting exception is the reduction of CA1 pyramidal layers, which matures with the hippocampus mainly postnatally in mice [56]. This coincides with onset of expression of the Cre-recombinase transgene in the AAC mice, driven by the mouse Thy1 gene promoter [31]. The identical CA1 defect in both strains of GSK3α-deficient mice not only confirm and strengthen each other, but imply that GSK3α did not impact importantly on any other brain-region during embryonal development. The specific neuroanatomical defect in CA1 caused by GSK3α deficiency thereby exemplify the fact that the GSK3 isozymes do not compensate for each other. A similar CA1 defect was observed in mice with neuron-specific deficiency of GSK3β [23] but only when they grow old (H. Maurin, B. Lechat, P. Borghgraef, F. Van Leuven; data not shown). Consequently, while GSK3α contributes to the postnatal development of the CA1 subregion, GSK3β could be more important for the ageing CA1. Conversely, both GSK3 kinases are activated by amyloid by increased tyrosine phosphorylation [29], and both can contribute to neurodegeneration. These important issues must be analyzed further, given the vulnerability of CA1 in AD [57,58].

The combined electrophysiological and cognitive defects of neuron-specific GSK3α deficient mice imply more important physiological functions of GSK3α in plasticity, acting both pre- and post-synaptically, than anticipated. The CA1 defective neuroanatomy is complemented by defects in hippocampal LTP and in cognition, which considerably extends observations on defective learning in a similar, independent total GSK3α-deficient mouse strain [21]. Although no motor impairments was observed in our GSK3α deficient mice, the cross with Tau.P301L mice led to severe motor problems on rotarod, and overall reduced mobility. Because GSK3α did not negatively affect motor performance, the large data-sets from the various tasks strengthen the observed impairments of memory and learning.

Overall, neuronal GSK3α contributes more subtly to behavior and cognition, judged from mice with neuron-specific deficiency. In contrast, total lack of GSK3α caused significantly more defects and impairments in more tasks and for more parameters, with increased anxiety as extra noteworthy.

### GSK3α phosphorylates protein Tau in brain in vivo

We focus on physiopathological roles of both GSK3 kinases in experimental AD-models by investigating contributions to amyloid pathology [23,29] and to the co-morbid tauopathy [29,35,36]. In our hands, neither GSK3 isozyme appreciably affected the production of amyloid peptides in mouse brain in vivo [23], while we have validated GSK3β as effective Tau-kinase [26,27,29,36]. Data presented here demonstrate physiological and pathological contributions of GSK3α to the phosphorylation of protein Tau, respectively of endogenous wild-type mouse Tau and of human Tau.P301L. In both instances, GSK3α acts effectively as Tau-kinase in vivo, although its quantitative contribution appears less than that of GSK3β. In translational terms, these data do not warrant the search for specific GSK3β inhibitors that spare GSK3α, as a therapeutic means for tauopathies in which GSK3 are thought to contribute.

### GSK3 is phosphorylated by cognitive training

While phosphorylation of S21/S9 in GSK3α/β respectively, is a negative control, the precise mechanism and effects of tyrosine phosphorylation at Y279/Y216 remain enigmatic. The presumed auto-phosphorylation in either GSK3 isozyme, increases their kinase activity and releases the otherwise stringent requirement for substrate-priming by other kinases. These are a prerequisite for effective phosphorylation by GSK3 of specified proline-dependent substrate sites [6]. The combined serine and tyrosine phosphorylation appears contradictory, although S21/S9 phosphorylation is accepted as overriding inhibiting parameter. We conclude that cognitive training in the paradigms described, inhibits the activity of both GSK3 kinases in the brain of wild-type mice by the demonstrated increased phosphorylation of S21/S9 in GSK3α/β.

## Conclusions

GSK3α activity affect not only the phosphorylation of protein Tau, but also impacts widely on motor activity and clinical parameters, although the repercussions are complex and more subtle than anticipated. These conclusions fit the notion that both GSK3 isozymes exert kinase activities on many protein substrates of varied identity, surpassing protein Tau as a microtubule binding protein. Structural and motor proteins, e.g. kinesins, tubulins, adapter proteins and others, are regulated by different types of phosphorylation, and several are known or suspected substrates for either or both GSK3 kinases. Our biochemical results on the impact of learning and memory on the phosphorylation of both GSK3 isozymes, and on the levels of protein Tau, need to be examined further in wild-type and informative transgenic mice.

## Methods

### Generation of mice with neuronal GSK3a deficiency

Mice with floxed GSK3α alleles [30] were crossed with Thy1-Cre recombinase transgenic mice [31]. Offspring was genotyped by PCR and qPCR on genomic DNA isolated from tail biopsies to define wild-type, floxed and recombined GSK3α alleles (see Results for details). Primers: 5′CCCCCACCAAGTGATTTCACTGCTA3′ and 5′AACATGAAATTCCGGGCTCCAACTCTAT3′. We generated neuron-specific GSK3α-deficient mice denoted as AAC and mice with floxed GSK3α genes but lacking Cre-recombinase were used as controls (AA-). Mice with total knock-out of GSK3α (GSK3α.KO) were generated and also analyzed. We further generated GSK3α.KOxTau.P301L bigenic mice to analyze the pathological phosphorylation of Tau.P301L protein by GSK3α.

### Biochemical analysis

Mice of either gender; as specified with the results, were anesthetized (Nembutal; 100 mg/kg, i.p), perfused transcardiacally for 2 min with ice-cold saline and the brain rapidly removed. Eventually, as indicated with the results, brains were placed on ice-cold glass plates for dissection of hippocampus and forebrain. Tissues were snap-frozen in liquid nitrogen and stored at −70°C until homogenization. Total homogenates were prepared in 25 mM Tris, 150 mM NaCl, 1× complete proteinase cocktail, 1 mM PMSF, 1 mM EGTA, 1 mM EDTA, 30 mM NaF, 1nM okadaic acid, 0.2 mM Na_3_VO_4_, 5 mM Na_4_P_2_O_7_ (pH 7.6). We used 6 ml buffer (ice-cold) per gram frozen tissue for homogenization with a cooled glass-teflon Potter-Elvejhem type of homogenizer (20 strokes, 700 rpm). Total homogenates (TH) were obtained after brief centrifugation (14000 g, 10 min, 4°C) to remove debris, and samples were stored in aliquots at −25°C until analysis [35,59,60].

Proteins were denatured, reduced and separated on 10% Tris-Glycine SDS-PAGE gels (Anamed, Germany). Proteins were transferred to nitrocellulose membranes and, after blocking, probed overnight (4°C) with primary antibodies: for both GSK3 proteins (Biosource, #44-610), for GSK3α/β pY279/216 (Biosource, #44-604) and GSK3α/β pS21/9 (Cell Signalling, #9331); total tau and phosphorylated tau were detected with Tau5 (Pharmingen, #556319), AD2 (Biorad, #56484), pT231 (Invitrogen, #355200), pS199 (Invitrogen, #44734G). Membranes were rinsed in TBS-Tween 0.1% and incubated with appropriate HRP-labeled secondary antibody diluted in 5% skimmed milk-powder in TBS-Tween 0.1%). Reactions were developed by chemiluminescence (ECL-prime; GE Healthcare) followed by digital picture acquisition and analysis (LAS 4000; ImageQuant v7.0; GE Healthcare).

### Immunohistological analysis

Anesthetized mice (Nembutal; 100 mg/kg, i.p.) were perfused transcardiacally with ice-cold saline and the brain rapidly removed. One hemisphere was immersion-fixed overnight in 4% paraformaldehyde in phosphate-buffered saline (PBS) at 4°C, rinsed, and stored at 4°C in PBS containing 0.1% sodium azide. Sagittal free-floating vibratome sections (40 μm) were cut and kept in 24 well plates, in PBS containing 0.1% azide at 4°C.

Immunohistochemistry was performed as previously described [29,35,60,61]. After rinsing in PBS, sections were pretreated for 15 min with 1.5% H_2_O_2_ in 50% methanol/PBS to eliminate endogenous brain peroxidase activity. Subsequent blocking of nonspecific binding sites by incubation in 10% fetal calf serum, 0.1% Triton X-100 in PBS (blocking solution), was followed by incubation at 4°C overnight with rabbit polyclonal GSK3 primary antibodies: GSK3α (Cell Signaling, #9338) and GSK3β (Cell Signaling, #9315). After rinsing (0.1% Triton X-100 in PBS), sections were incubated for 1 h with the biotinylated goat anti-rabbit secondary antibody, diluted 1:500 in blocking buffer. Sections were further incubated for 30 min with avidin-biotin complex (Vectastain ABC Elite, Vector labs). Sections were rinsed in PBS and incubated for 5 min in 50 mM Tris–HCl (pH7.6), before staining with 3,3′-diaminobenzidine (0.5 mg/ml), 0.3% H_2_O_2_ in 50 mM Tris–HCl (pH 7.6). Mayer’s hematoxylin (Sigma, #51275) was used for counterstaining, prior to dehydration by passage through a graded series of ethanol-water mixtures. After two washes in 100% xylol, the dehydrated sections were mounted using (DePeX) [60].

### Hematoxylin staining

Free-floating sections were incubated overnight at 4°C in PBS-TX100 (0.1%), washed 3 times in PBS before being mounted on silanized glass-slides. The sections were allowed to dry for 2 hours at 50°C before staining with Mayer’s Hematoxylin (Sigma, #51275) followed by dehydration in the classical graded ethanol/water series. After two washes in 100% xylol, the sections were mounted (DePeX). The area of the CA1 pyramidal layer was measured on 3 sections per mouse, spaced 120 μm apart, by digital image analysis and expressed relative to the entire CA1 area, measured on the same sections.

### Breeding performance and sperm analysis

Breeding performance for all genotypes was estimated from the number of litters and pups produced in our breeding colony. Sperm analysis was essentially as described [62]: cauda epididymis was dissected and minced in 500 μL of in-vitro fertilization IVF medium (Minimum Essential Medium supplemented with 0.025 mg/ml sodium pyruvate, 0.07 mg/ml penicillin, 0.05 mg/ml streptomycin, 3.8 μg/ml EDTA and 3 mg/ml BSA) and kept at 37°C for 10 min. The cell suspension was analyzed by the swim-up test: 100 μL was diluted with 500 μL warm medium and incubated at 37°C for 15 min. The upper and lower fractions were separated and cells counted in Neubauer chambers. Relative sperm motility was calculated as the ratio of spermatozoids in the upper and lower fractions.

### Behavior

Behavioral experiments were designed to allow performance in consecutive tasks without interference, with stressful tasks, e.g. footshock threshold, assessed in distinct groups of mice. We always analyzed age- and gender-matched AA- and AAC mice (n=11 or 12; except for beamwalk and grid hanging tests with n=5). Behavioral tasks were assessed in FvB females (n=9), Tau.P301L females (n=10), GSK3α.KO females (n=6) and GSK3α.KOxTau.P301L females (n=8). Experiments were performed over a total time-span of 8 weeks to minimize interference between tasks.

#### Rotarod

Motor ability and coordination was assessed by the classical rotarod [35]. Mice were trained for 3 days in 3 sessions per day (3× 3 min at 20 rpm). Test-trials were performed on day 4, during 5 min with the rod rotating at increasing speed (4 to 40 rpm). Data are expressed as the time the mice remained on the rod.

#### Inverted grid test

Performed with a grid (425×266 mm, 1291 Eurostandard type III H, Tecniplast) kept at 40 cm above the bench that was covered with soft tissues. Data are expressed as the time in seconds, until the mice released their grip after inversion of the grid (maximum 60 sec) [26].

#### Beam-walk

The test was performed by placing the mouse in the middle of a horizontal rod (diameter 1 cm, length 1 m) placed 35 cm above the bench that was covered with soft tissues. Data are expressed as the time spent on the rod (maximum 3 minutes) [35].

#### Open field test

Mice were placed in a corner of a perspex box (52×52×40 cm) with black walls and translucent floor, dimly illuminated from underneath. The activity of individual mice over a 10 min observation period was recorded and analyzed by a dedicated system (EthoVisionXT 7.0 Noldus, Wageningen, The Netherlands). Calculated parameters included total distance, speed, time spent in defined sections [29].

#### Y-maze

Mice were placed in one arm of the Y-shaped maze constructed from opaque plastic, and allowed to explore the 3 arms for 6 min. The number of entries in each arm, and the alterations were recorded. Entry was considered effective when all 4 limbs were located inside the arm [44].

#### Dark/light test

This task was performed in the same perspex box as open field test, now divided in two compartments: one remaining open and illuminated from above, and one darkened by covering with a fitting box made of grey opaque plastic. Mice were initially placed in the dark compartment and observed for 5 min by recording the time spent in each compartment and the number of transitions between dark/light compartments [29].

#### Passive inhibitory avoidance task (PIA)

This task was performed in a two-chambered inner box comprising lit and dark sections separated by a trap-door, all placed inside a larger sound-tight box. For training, mice were initially placed in the lit section and after 10 sec the trap-door was opened to allow the mouse to enter the dark section, with an electric foot-shock (0.5 mA; 2 sec) delivered after 2 sec. The mice were kept for 15 sec in the dark compartment before being returned to the home cage. Retention was assessed 24 hours later, by placing the mouse in the lit section again and measuring its latency, i.e. the time that elapsed before entry into the dark compartment [29].

#### Novel object recognition task (NORT)

NORT was performed as described [31]. Briefly, mice were habituated for 10 min to the perspex open-field box, dimly illuminated from below. On day 2 the mice were observed in the same box for 8 min in the presence of object A (acquisition). The time that the mouse explored object A was recorded, with the criterion that the snout was within 1 cm of the object. The 8 min retention trial was performed 4 hours later, by placing the mouse in the box with the additional novel object B. The time that the animal spent exploring both objects was recorded as tA and tB, respectively, and the retention index (RI) was defined as tB/(tA + tB) × 100.

#### Porsolt forced swim task

The original test [42] was adapted by placing the mice in a large glass beaker of 1 liter (diameter 10 cm) filled with 800 ml water (23°C) that was changed after each individual test. Mice were allowed to habituate for 2 min followed by 4 min observation with recording the time of immobility and passive floating.

#### Footshock sensitivity

Mice were placed in the perspex box used for PIA with metal wire floor and allowed for 2 min to habituate before receiving an electric foot-shock (0.1 mA, 1 sec). Subsequently, the mice received every 30 sec an electric foot-shock with an increment of 0.05 mA until 0.6 mA and of 0.1 mA until a maximum of 1 mA. The voltage at which the mice reacted by flinching, jumping or vocalizing was recorded [63].

### Electrophysiology

Electrophysiological analysis was performed as described [14]. Acute hippocampal sections (400 μm) were kept in artificial cerebrospinal fluid (ACSF) 124 mM NaCl, 5 mM KCl, 26 mM NaHCO3, 1.24 mM NaH2PO4, 2.5 mM CaCl2, 1.3 mM MgSO4, 10 mM glucose aerated with 95% O2/5% CO2. Sections were placed on the interface recording chamber and kept at 28°C for 90 min under perfusion with ACSF (1 ml/min). Bipolar twisted nickel-chrome electrodes were used to stimulate Schaffer’s collaterals, while extracellular field excitatory postsynaptic potentials (fEPSP) were recorded in the CA1 stratum radiatum using low resistance glass electrodes filled with ACSF. LTP was induced by strong stimulation, 4 trains at 100 Hz of 1 s with 5 min intervals, with subsequent measurement of the potentiated response. The slope of fEPSP was averaged over 4 consecutive responses and normalized to the mean value of baseline recorded for 30 min prior to stimulus. For each slice the input–output curve was established by increasing stimulus intensity and synaptic fatigue was measured by recording the output during high frequency stimulation. Paired-pulse facilitation was determined at four different inter-pulse intervals: 50 ms, 100 ms, 150 ms and 200 ms, and measured by the relative ratio of the second to the first fEPSP slope.

### Statistical analysis

All data are expressed as mean±SEM. The statistical tests were performed with dedicated software (Graphpad Prism 5.04). Behavioral data (Tables 1 and 2) were analyzed by Student’s t-Test (unpaired, two-tailed). Other datasets were analyzed by one – or two-way Anova with appropriate post-hoc test, as specified in the legends. Classical levels of statistical significance are indicated with the data.

### Animal healthcare

All experiments were performed in accordance with regional, national and European regulations concerning animal welfare and animal experimentation, and were authorized and supervised by the University animal welfare commission (Ethische Commissie Dierenwelzijn, KULeuven).

All animal experiments were performed by certified researchers conforming to regional, national and European regulations concerning animal welfare and animal experimentation, authorized and supervised by the university animal welfare commission (Ethische Commissie Dierenwelzijn, KULeuven). We formally declare that we comply to the European FP7-Decision 1982/2006/EC, Article 611, i.e. all research activities is carried out in compliance with fundamental ethical principles and all experiments are approved and overlooked by the Animal Welfare Commission.

## Abbreviations

ACSF: Artificial cerebrospinal fluid; AD: Alzheimer’s disease; CA1: Cornus ammonis 1; fEPSP: Field excitatory postsynaptic potential; GSK3: Glycogen synthase kinase 3; LTP: Long term potentiation; NORT: Novel object recognition task; OFT: Open field test; PIA: Passive inhibitory avoidance; PPF: Paired pulse facilitation; TH: Total homogenate; TRPV1: Transient receptor potential cation channel subfamily V member 1.

## Competing interests

The authors declare no financial competing interests.

## Authors’ contributions

FVL originated the concept and overall design of the study. IDW and LR performed the electrophysiological analysis. BL, HM and JVL monitored breeding, performed genotyping and analyzed behavior. PB and HM performed immunohistochemistry and analyzed data. BL and HM performed biochemical analysis and sperm analysis. BL, HD, HM and TJ performed general characterization of mice. HM, BL, IDW and FVL wrote the manuscript. All authors approved the final manuscript.
